# Neuroprotective Surgical Strategies in Parkinson’s Disease: Role of Preclinical Data

**DOI:** 10.3390/ijms18102190

**Published:** 2017-10-20

**Authors:** Napoleon Torres, Jenny Molet, Cecile Moro, John Mitrofanis, Alim Louis Benabid

**Affiliations:** 1 University Grenoble Alpes, CEA, LETI, CLINATEC, MINATEC Campus, 38000 Grenoble, France; jenny.molet@cea.fr (J.M.); cecile.moro@cea.fr (C.M.); alimlouis@sfr.fr (A.L.B.); 2 Department of Anatomy, University of Sydney; Sydney Medical School, Sydney NSW 2006, Australia; john.mitrofanis@sydney.edu.au

**Keywords:** neuroprotection, Parkinson disease, surgery, cell grafts, glial neurotrophic derived factor, deep brain stimulation

## Abstract

Although there have been many pharmacological agents considered to be neuroprotective therapy in Parkinson’s disease (PD) patients, neurosurgical approaches aimed to neuroprotect or restore the degenerative nigrostriatal system have rarely been the focus of in depth reviews. Here, we explore the neuroprotective strategies involving invasive surgical approaches (NSI) using neurotoxic models 1-methyl-4-phenyl-1,2,3,6-tetrahydropyridine (MPTP) and 6-hydroxydopamine (6-OHDA), which have led to clinical trials. We focus on several NSI approaches, namely deep brain stimulation of the subthalamic nucleus, glial neurotrophic derived factor (GDNF) administration and cell grafting methods. Although most of these interventions have produced positive results in preclinical animal models, either from behavioral or histological studies, they have generally failed to pass randomized clinical trials to validate each approach. We argue that NSI are promising approaches for neurorestoration in PD, but preclinical studies should be planned carefully in order not only to detect benefits but also to detect potential adverse effects. Further, clinical trials should be designed to be able to detect and disentangle neuroprotection from symptomatic effects. In summary, our review study evaluates the pertinence of preclinical models to study NSI for PD and how this affects their efficacy when translated into clinical trials.

## 1. Introduction

Parkinson’s disease (PD) is a progressive disease that damages neurons mainly in specific dopaminergic brain areas, producing debilitating signs, such as resting tremor, akinesia, and rigidity [1]. These primary motor signs, if lateralized, can be clinically diagnostic of PD. However, they are only a subset of the mixed motor, cognitive, affective, autonomic, and even sensory deficiencies that result from the selective degeneration of other neuronal types [2]. The first line of treatment is dopamine replacement drug therapy, which, although providing initial treatment of the signs of the disease, shows declining efficacy over time and leads to pronounced side-effects in later stages. Some non-motor symptoms can also produce complications in the management of the disease in the later stages. More recently, various surgical interventions have been developed to alleviate the motor signs of the disease; these are recommended once the efficacy of the drug therapy subsides and the side-effects develop [3,4]. However, none of the current therapies have been shown to convincingly slow the progression of PD. This is because, in part, there are considerable methodological difficulties in determining whether an agent is neuroprotective [5].

Therapeutic strategies that slow or stop the neurodegenerative process of PD are considered to have a major impact on the evolution and further development of PD. Neuroprotective drugs or compounds have been the focus of a large number of publications in recent years, although only a few have progressed to clinical trial. Indeed, there have been several major reviews considering these issues in order to discover disease modifying drugs for use in humans [6,7,8]. Unfortunately, drug therapies have been largely unsuccessful and none have been approved for use as a neuroprotective agent [9]. Drugs have a systemic effect and need to pass the blood brain barrier undisturbed to reach target areas in the brain; furthermore, these drugs may impact on other organs, thereby increasing the possibility of side effects. In this context, there is a strong case for the local delivery of therapeutic agents via stereotactic neurosurgery, and such methods may well have a role in neural protection and/or restoration in PD. In this review, we will focus on the neuroprotective strategies involving invasive surgical approaches (NSI), their corresponding preclinical assays and the resulting clinical data in order to evaluate the efficacy of these strategies to modify the progression of PD.

For clarification proposes, and, according to the operational definition of the CINAPS (Committee to Identify Neuroprotective Agents in Parkinson’s disease), in this article, neuroprotection will be considered any intervention that favorably influences the disease process or underlying pathogenesis to produce enduring benefits for patients. Indeed, this definition comprises related terms such as “neurorescue”, “neurorestoration” and “disease-modifying” [7].

## 2. Results

### 2.1. Neuroprotection Secondary to Deep Brain Stimulation of the Subthalamic Nucleus

Deep Brain Stimulation (DBS) is the most rapidly expanding field in neurosurgery. In the early 1950s, soon after the establishment of human stereotaxic surgery, chronic stimulation of subcortical structures was used [10]. In 1987, Benabid and co-workers introduced chronic DBS of the thalamic nucleus ventralis intermedius as a therapeutic strategy in PD [11]. In the 1990s, with an increase in the understanding of the pathophysiology and involvement of the basal ganglia in movement disorders [12], many authorities started using the subthalamic nucleus (STN) as a surgical target. Indeed, animal models of PD have played a crucial role in the discovery of the STN DBS therapy [13,14].

Since its discovery, STN DBS has become a standard alternative for the treatment of advanced PD, leading to prominent improvements in motor function and quality of life of PD patients, particularly after the efficacy of dopamine drug therapy has declined. The STN is a key node in the functional control of motor activity in the basal ganglia. The dopamine loss that occurs in PD augments STN activity, but its inhibition by DBS suppresses the motor in both human patients and animal models of PD [15].

It has been proposed that this augmented STN activity may, in turn, cause further damage to vulnerable dopaminergic neurons in the substantia nigra pars compacta (SNc.) There is a small but distinct glutamate output from STN to the SNc, which may contribute to the neurotoxic process underlying dopaminergic cell death in PD. This circuit could underlie a scenario for an increasing cycle of neuronal loss in the SNc [16]. DBS inhibits the STN neurons and is likely to result in the suppression of their glutamate output, hence reducing glutamate neurotoxicity and death [17]. There is also evidence that high-frequency activation of glutamatergic synapses triggers the release of BDNF, a protein brain-derived neurotrophic factor, which is able to protect neurons from degeneration [18]. In the section that follows, we will review the preclinical and clinical evidence of neuroprotection secondary to STN DBS.

#### 2.1.1. Preclinical Data

There was some early evidence of dopaminergic cell protection by using methods other than DBS, in particular methods for “silencing” STN, most notably ablation of the nucleus [19,20]. However, even if the effects of chronic DBS stimulation are comparable to an STN lesion, it is essential to explore chronic DBS in laboratory animals in order to investigate the hypothesis that there is neuroprotection in the SNc after DBS of the STN in PD. Five papers have reported the neuroprotective properties of DBS of the STN in vivo, either in 6-hydroxydopamine (6-OHDA)-lesioned rats [21,22,23,24] or 1-methyl-4-phenyl-1,2,3,6-tetrahydropyridine (MPTP)-treated non-human primates (NHP) [25]. All have found neuroprotection of the dopaminergic phenotype comparing the treated side vs. controls (between 15% and 48% absolute neuroprotection).

The earliest studies on neuroprotection in PD using chronic DBS were performed in 2004 by Maesawa et al. in 6-OHDA-lesioned rats [21]. These authors used externalized equipment and stainless steel electrodes, due to the smaller size of the experimental animal. The authors observed 48% neuroprotection in the stimulated side using continuous stimulation applied at the same time of the lesion. Another group, using fractionalized (1 h/day) stimulation and clinical grade electrode (Pt/Ir), found 25% neuroprotection [22]. The equipment was also externalized and the treatment was applied after seven days of exposure to 6-OHDA. In order to mimic the clinical situation more closely, recent papers used totally implantable equipment and clinical grade Pt electrodes; for example, Harnack et al. (2008) [23], and Spieles-Engemannzt et al. (2010) [24] in rats and Wallace et al. [25] in monkeys. These authors also delayed treatment until the lesion was well established, to better mimic the time course of PD (5–15 days post lesion). Despite the different methodologies, all studies produced histological evidence of nigral dopaminergic neuroprotection, and some evidence of behavioral recovery in DBS-treated animals [21]. Some authors have even found significant levels of BDNF (a neuroprotective endogenous compound) in the nigrostriatal system after unilateral STN DBS [26]. Our systematic review did not yield any study reporting a negative effect of DBS of the STN in PD. However, there has been a very recent study that assessed the neuroprotective potential of DBS of the entopeduncular nucleus, the homologous structure to the internal part of globus pallidus (GPi) in rats, and found no histological evidence of nigral protection or levels of neuroprotective factor (BDNF levels), nor amelioration of motor impairments [27].

#### 2.1.2. Clinical Data

The evaluation of neuroprotection of the STN after DBS has proven to be very problematic in the clinical situation. Potentially neuroprotective effects can be challenged, mainly because the symptomatic effects of DBS of the STN are very difficult to disentangle from the potential protective effects. Here, in this review, we considered only the long-term evaluation studies (>4 years) that also have a proper off medication/off stimulation evaluation and identified 12 studies that cover the set criteria (Table S1). All of the clinical trials reported symptomatic positive results, even when compared with the best medical therapy, but did not precisely address the potentially neuroprotective effects. Only Merola et al. [28] have investigated the comparative potential disease modifying effect of DBS of the STN vs. best medical therapy. They found a similar progression of motor score and cognitive/behavioral alterations in both groups with no reduction in the motor scores during off medication/off stimulation evaluation. In general, in all of the other studies, the evaluation during off stimulation/off medication after >4 years did not show any clear disease-modifying effects over motor score. Three articles showed a deterioration of motor symptoms: Zibetti et al. and Gervais-Bernard et al. found worsening motor scores (7%) after a five-year follow-up and Visser-Vandewalle et al. found a non-significant 26% worsening after four years [29,30,31]. The rest of the studies found a stable motor score or non-significant improvement after >4 years of STN [28,32,33,34,35,36].

One study, not included in our review criteria, but specifically addressing the subject of disease modification, was performed by Hilker et al. [37]. In 30 patients with successful STN DBS, using 18F-fluorodopa (F-Dopa) positron emission tomography (PET), these authors found annual PD progression rates of 9.5–12.4% in the caudate and 10.7–12.9% in the putamen. These values are within the range of data reported previously from non-stimulated PD patients, suggesting a similar evolution in both cases [37].

### 2.2. Glial-Cell-Line-Derived Neurotrophic Factor (GDNF) Therapy and Neuroprotection

Glial-cell-line-derived neurotrophic factor (GDNF), a glycosylated disulfide-bonded homodimer, promotes mesencephalic dopaminergic neuronal survival in several in vitro and in vivo models [38]. With the degeneration of dopaminergic neurons being a major contributor to the development of PD signs, GDNF appeared to be a promising therapeutic agent. However, this neurotrophin has been shown to be unable to cross the blood–brain barrier, complicating its clinical use immensely. Therefore, ways to deliver GDNF effectively into the central nervous system are needed.

#### 2.2.1. Preclinical Data

We found 28 preclinical studies investigating the putative neuroprotective properties of GDNF. Several administration routes were used, from early systemic injection, to intraventricular and intraputaminal, and finally using viral vectors to deliver GDNF or neurturin (NTN). In the section that follows, we focus on the preclinical data supporting the clinical trials.

For the intracerebroventricular administration of GDNF, we found 11 papers corresponding to our filtered criteria in rats and primates. In 6-OHDA-lesioned rats, seven papers demonstrated behavioral and histological recovery; on the whole, these studies showed an increase in dopamine content and increase in dopaminergic cells in the SNc, but no positive restoration in the striatum [38,39,40,41,42,43,44]. For example, Lapchak et al. (1997) reported that either intranigral or intraventricular GDNF administration is capable of producing behavioral and histological changes in 6-OHDA-lesioned rats [40,45]. These authors also showed that, in aged rats, there is an increase in locomotor and dopaminergic activity after intraventricular GDNF [46]. Only one study reported no effect following intracerebroventricular GDNF [47], and none reported a toxic effect in rats. In MPTP-treated monkeys, although there has been some minor weight loss, the bulk of the studies indicate positive outcomes (four papers), and no negative ones [48,49,50,51]. GDNF was shown to improve dopaminergic function in the SNc, although not in the putamen of unilateral MPTP-treated monkeys [49], and intraventricular GDNF showed low affinity for the caudate and putamen [52].

Regarding the intraputaminal route, we found 11 papers containing pertinent preclinical data. Eight papers had positive outcomes: behavioral and histological restoration of function was seen in the SNc and striatum of MPTP-treated mice [53], 6-OHDA rats [47,54,55,56] and MPTP-primates [57,58]. In contrast, three articles presented negative outcomes in the 6-OHDA-lesioned rat model of intrastriatal GDNF injection. Rosenblatt et al. showed the almost complete protection of SNc cell bodies, but not of their dopaminergic (tyrosine hydroxylase) phenotype nor their striatal re-innervation. Consistent with these data, behavioral changes were not seen after treatment [42,59]. Further, Kirik et al. described that when GDNF was infused in the striatum, motor behavioral improvements were transient and subsided shortly (few days) after GDNF delivery [44].

A third approach also explored the use of a viral vector to deliver GDNF in preclinical studies. The adeno-associated virus (AAV) produces durable expression GDNF in target areas (striatum injection). We recorded eight positive reports, using rats and monkey PD models. In five studies in 6-OHDA-lesioned rats, a 200–600% increase in the number of dopaminergic cells has been reported, 5–6 months after a single injection [60,61]. Results were less impressive when AAV-GDNF injection was administered four weeks after 6-OHDA lesion, but still positive (70% increase in dopaminergic cell number) [61]. Other works in NHP, one in aged monkeys [62] and three in MPTP-NHP [63,64,65], described positive histological changes in the striatum and the SNc, as well as behavioral improvements with no side effects. There are no negative reports for this precise delivery approach.

#### 2.2.2. Clinical Data

We found seven clinical studies using GDNF, each with different routes of administration (Table 1). The first study concerned safety and tolerability, in which a PD patient received monthly intracerebroventricular injections of GDNF. Unfortunately, these injections led to continued worsening of his PD signs, several side effects and no evidence of nigrostriatal regeneration [66]. This work was followed by a clinical trial by Nutt et al. in 2003 [67], who performed a double-blind multi-centric, placebo-controlled assessment of safety, tolerability, and biological activity of GDNF administered by an implanted intracerebroventricular (ICV) catheter in advanced PD. As many as 50 subjects were enrolled in this clinical trial. Their findings indicated no clinical improvement in the patients, notwithstanding GDNF being found to be biologically active. In recent times, some concern has been raised about the dosage and the diffusion of GDNF, factors that could have influenced the results of the clinical trial [67]. Other studies adopted a change in the administration route, to direct intraputaminal infusion. This change resulted in positive outcomes. In 2003, Gill et al. studied five patients and found a reduction in the off medication score and a clear reduction (64%) of medical-induced dyskinesia, with no side effects. Moreover, 18F-Dopa PET neuroimaging, revealed a direct effect of GDNF on dopamine function by generating a significant increase in putamen dopamine storage after 18 months [68]. Slevin et al. in a larger cohort (*n* = 10), reported equivalent results with unilateral intraputaminal GDNF perfusion and no side effects at the six-month and one-year follow-ups [69,70]. These encouraging results prompted a large multi-centric randomized controlled trial using intraputaminal bilateral infusion of GDNF. A cohort of 34 patients was enrolled in the study. However, no statistical differences in the Unified Parkinson’s Disease Rating Scale (UPDRS) off motor score (part III) were found in the treated and non-treated patients. Serious device-related side effects and antibodies against the compound completed the overall negative outcome of this study [71].

As the administration route was associated with the negative results of this controlled trial, another, different approach was proposed by Marks et al. in 2008 [72]. A member of the GDNF family, neurturin (NTN) was injected using gene delivery via AAV vector. They performed intraputaminal bilateral injection of adeno-associated virus serotype 2-neurturin (CERE-120) in twelve patients, resulting in 34% improvements in off medication UPDRS-III, with no significant side effects [72]. This same group completed the evaluation with a multi-centric double-blind, phase 2 randomized trial. The results were again negative, however, with the CERE-120 bilateral infusion being not significantly different from sham surgery. Finally, adverse effects were seen in 13 out 38 patients and three developed brain tumors [73].

### 2.3. Cell Grafting or Transplant-Based Therapies and Neuroprotection

The idea that a transplantation of immature, embryonic dopaminergic neurons could promote structural and cellular restoration of basal ganglia degenerative pathways seen in PD is potentially a good one. Grafts could replace the lost dopaminergic neurons in patients with PD [74]. Following successful transplant therapies in the mid-1980s, a surge of clinical trials using different approaches occurred started in the 1990s. We describe here key animal experiments and also open label clinical trials and major controlled trials that have been published during the past 40 years.

This review has identified several neuroprotective strategies implicating invasive approaches (NSI) requiring grafting cells of different origin. Historically, the first attempt at transplantation was the autologous adrenal transplantation into the striatum. In the suprarenal gland, adrenal chromaffin cells produce more epinephrine than dopamine; however, when isolated adrenal chromaffin cells are grafted into brain tissue, an increase in dopamine expression has been shown [75]. A second approach was the use of embryonic dopaminergic cells. The grafts taken from the embryonic ventral mesencephalon can be removed following their last mitosis and prior to completing axonal connections. These embryonic cells may then produce connections with the host to re-establish dopaminergic innervation [76]. More recently, two NSIs have been attempted. One used a carotid body graft, in which intrastriatal transplantation of dopaminergic carotid body (CB) cells is undertaken; these cells have high dopamine content and exert trophic effects [77,78]. Finally, autologous bone-marrow-derived mesenchymal stem cells (hMSCs) have also been used to restore nigral striatal pathways. hMSCs have different multi-lineage capacity, which also includes the ability to differentiate toward the dopaminergic phenotype, and they are easily obtained through a safe procedure (bone marrow aspirate). Moreover, they have the capacity to migrate to lesioned areas and release factors capable of modulating the inflammatory response, inducing neuroprotection [79,80].

#### 2.3.1. Preclinical Data

Adrenal transplantation into the striatum has been reviewed extensively elsewhere [75]. In brief, the technique produced mixed results in preclinical studies. Adrenal chromaffin cells survive poorly when grafted into the striatum, due primarily to the low levels of endogenous growth factor within the structure [81,82]. In addition, the presence of non-chromaffin constituents of the graft (fibroblasts and endothelial cells) has been alleged to impair graft survival in a number of Central Nervous System (CNS) sites. Nevertheless, most of the studies have found behavioral improvements after the procedure, even though there were no accompanying [83] or very few tyrosine hydroxylase positive (TH+) cells within the graft [84], and less than 1% are able to release dopamine [85]. Studies in rodents have had more positive outcomes, with good graft survival after the removal of non-chromaffin constituents [86]. Several other strategies have been attempted in order to ameliorate graft survival with some success [84,87].

Embryonic dopaminergic cells have been used extensively in preclinical studies since the first successful transplantation in rodents. Our review identified 19 articles using primarily a rat model of PD, together with some key primate experiments (total of eight) that have guided clinical trials. The principal issue in the preclinical studies has been graft survival [75,88]. The survival rate of transplanted dopaminergic neurons is only in the range of 5–20% [89], with the vast majority of cells dying during the surgical procedure or within the first days post-surgery. To ameliorate the chances of survival, several strategies have been conceived, considerating the age of the graft donor [90], local application of growth factors [89,91], and the use immunosuppression agents [92] or the identification for the graft of special subtypes of dopaminergic neurons (GIRK2+) which are able to project to the dorsolateral putamen. These strategies have shown variable degrees of success. The particular subtypes of dopaminergic neurons have been shown to be more likely to innervate the striatum and result in a more “normal” nigrostriatal pathway. Hence, an important strategy could lie in the identification of those dopaminergic cell subtypes that are more likely to re-innervate the striatum before implantation [93]. Even though there is a limited survival rate in most of the studies, the few remaining grafted neurons appear to reverse many behavioral deficits evident in experimental animals, for example, apomorphine-induced rotation in 6-OHDA-lesioned rats [90]. Some behavioral deficits however, like skill forepaw, are relatively resistant to transplant-based therapy [94]. No toxicity from the preclinical studies is reported, while a major side effect of clinical trials, like levodopa-induced dyskinesia, is improved in rats after embryonic graft [95].

Carotid body (CB) and the hMSCs grafts have been attempted in order to search for new tissues sources of neurotrophic factors and dopamine. For CB, the preclinical efforts have been centered on 6-OHDA-lesioned rat and MPTP-treated primate models. Studies in rats have used a co-transplantation approach in which CB cells and ventral mesencephalic cells (VMC) are grafted simultaneously; these show positive functional and histological recovery [78]. In the primate studies, these used only unilateral striatal transplants of CB cells, and obtained the protection of nigral dopaminergic neurons on the ipsilateral side [96]. The hMSCs as a source of graft have also been used in several preclinical trials in rats (8) and in mice (1). These studies demonstrated that 6-OHDA-induced damage increases the viability of transplanted MSC and attracts these cells to the lesioned side [80]; further, there was amelioration of amphetamine-induced rotations [97], partial restoration of the dopaminergic markers [98], maintenance of striatal/nigral dopaminergic terminal survival and an enhanced neurogenesis in the subventricular zone (SVZ) [99], coupled with a reduction of the behavioral abnormalities. Offen et al. also showed positive outcomes in mice when most of the transplanted cells survived in the striatum and expressed TH. Some of the TH positive cells even migrated toward the SNc [100]. In contrast, a study by Camp et al., found that grafted cells provoke a marked immune response and did not prevent nigrostriatal dopamine depletion [101]. Taken together, these findings and approaches were the basis for clinical trials in patients with advanced PD.

#### 2.3.2. Clinical Data

Clinical data concerning the grafting of cells in PD patients dates back to early 1980s (Table S2). Our review of the literature revealed the use of adrenal autologous transplants in advance PD in several open label trials (*n* = 3) and one multi-centric open evaluation study. The first studies were conducted by Backlund (1985) and Lindvall (1987), who reported only transient motor improvements or no changes after six months to two years of follow-up; there was also a severe psychiatric side effect recorded in one subject [102,103]. These results were in contrast with the data from Madrazo et al., in which all patients presented immediate clinical improvement after implantation. In fact, the first patient obtained complete disappearance of the rigidity and akinesia 10 months after surgery [104]. Unfortunately, this encouraging result was not reproduced in the large multi-centric study (*n* = 61) by the United Parkinson Foundation Neuro transplantation Registry Group; this study found only a small 19% motor improvement in patients. Further, there was an 18% mortality, with half of the deaths related to surgery, and 22% of survivors had persistent psychiatric morbidity not present prior to surgery [105].

Embryonic dopaminergic cells are the preferred source of graft used in clinical trials, in order to restore circuitry and function in the lesioned brain of PD patients. The bibliographic review reported 15 open label studies, one randomized delayed onset and two randomized double-blind sham surgery studies. The open label trials have been revised in great detail in several review articles [106,107]. In summary, reduced bradykinesia and rigidity with minor effects in tremor were observed in patients. In addition, there is a better response to L-Dopa; in other words, a reduction in dose or even cessation of medication in some cases. Imaging studies using positron emission tomography have also reported on graft survival and function. Most of the open label studies showed no serious complications. However, Freeman et al. reported asymptomatic superficial cortical hemorrhage, transient postoperative confusion and hallucinations [108]. Moreover, delayed asymmetrical dyskinesia, and off-medication dyskinesia was only seen in two of the more recent open label studies [109,110]. Hence, the success of the initial open label studies was not matched by the results obtained in two large controlled trials. Indeed, Freed and co-workers analyzed the results in bilateral grafted putamen using self-assessment scales; in this case, no differences were noted between the non-grafted control patient and the grafted cohort at 12 months after surgery. PET examinations showed F-Dopa uptake increased in the graft recipient and in post mortem analysis, but a modest survival of dopaminergic neurons in the SNc. Dystonia and dyskinesia were present in 15% of the patients, three years after the graft; they persisted even after L-Dopa was discontinued [111]. Olanow et al. found no significant overall treatment effect UPDRS-III in off between groups 24 months after surgery and F-Dopa imaging increased in grafted groups at 24 months. Moreover, they reported graft-related dyskinesia in as many as 56% of their transplanted patients [112].

Carotid body (CB) and the hMSCs grafts have also been tested in clinical trials, based on good preclinical results and the broad acceptance of the stem cell source. Mínguez-Castellanos et al. [77] conducted an open label phase I-II trial in which they performed bilateral stereotactic implantation of CB cell aggregates into the striatum. They obtained a decrease in UPDRS-III scores at six months of 23%, 5–74% at one year and 15–48% at three years of follow-up. F-Dopa imaging of these patients revealed a non-significant 5% increase. Venkataramana et al. (2010) conducted an open label trial using hMSCs unilateral transplantation in the subventricular zone. They found that mean “off” score improved by 22.9% and mean “on” score by 38%, from the baseline at 36 months with no adverse effects. In this trial, 2 patients reduced L-Dopa doses [113]. These results encouraged the authors to continue with a second open label study in 12 patients, hMSCs bilateral implantation into the subventricular zone. The results were a mean improvement of 17.92% during “on” and 31.21% during “off” period on motor scales, less important than previous study. The patients with advance PD responded less well to the graft than early diagnosed patients [114].

## 3. Discussion

**Figure 1:**
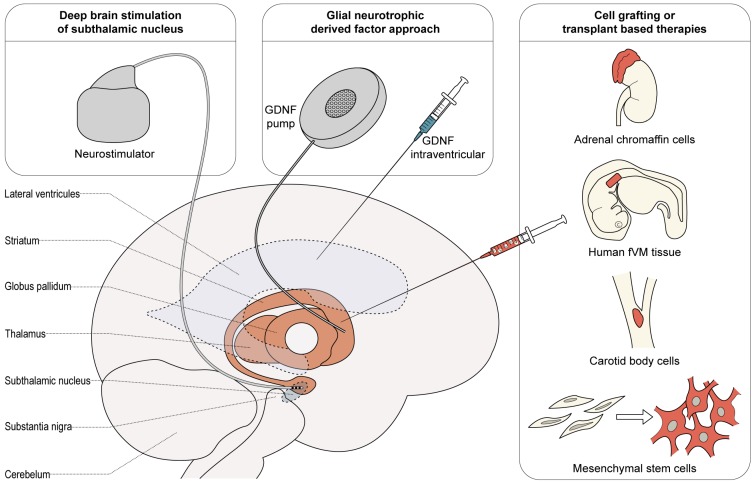
Different neuroprotective surgical strategies or interventions (NSI) for PD. All of the restorative therapies have been tested in animal models of PD and used in controlled clinical trials. PD: Parkinson’s disease; STN DBS: Subthalamic nucleus deep brain stimulation; GDNF: Glial cell line-derived neurotrophic factor; fVM: fetal ventral mesencephalic (see text for details).

In this review, we have identified three families of non-pharmacological, NSI, approaches targeting neuroprotection in PD (Figure 1). These approaches were tested in preclinical animal models, from rodents to monkeys, with positive outcomes for neuroprotection. All of the animal models used were toxin-induced, and mainly rodents. The use of large animal models (mainly MPTP-treated monkeys) was limited but present in most NSI arriving to clinical trials [25,83,115,116]. Interestingly, no testing has been done in α-synuclein or other genetic models of PD.

In general, the procedures and devices were down-sized in most of the preclinical animal models, to better facilitate size and anatomical differences of the animal models (i.e., DBS electrodes specifically developed for primates), before translation into clinical trials. There were very few to no adverse effects reported during the preclinical phase in contrast to clinical studies. Hence, the preclinical models used appear not to be good indicators for adverse events and/or side effects found in clinical trials (i.e., off-medication dyskinesia after embryonic cell transplantation).

A consistent feature of the different NSI approaches is that, despite showing promise at the preclinical level, each has failed to show convincing clinical efficacy as neuroprotective agents. For each approach, these failures appear to have been for different reasons. For DBS of the STN, secondary neuroprotection may not have been evident clinically because of methodological and technical limitations producing certain difficulties in the analysis of neuroprotection, and disentangling it from symptomatic effects. For GDNF, infusion in striatal areas generally failed to show the degree of efficacy and new preclinical evidence raised questions about the long-term security of the agent (cerebellar degeneration in a series of monkeys) [117]. For cell grafting or transplant-based therapies, there were low survival rates in the transplanted cells and no clinical benefit found [111], leading to a loss of interest in the field [112].

From the analysis of different results, we can see the importance of how to increase the predictive and translational value of preclinical animal studies in this field. Animal models of PD have played a crucial role in the discovery of the new therapies associated with this and most other diseases. The best preclinical models need to reproduce as many aspects of the disease as possible—from histological to clinical features—to have a critical indication of the outcome and toxicity in clinical trials. Therapeutics can be directed towards the alleviation of symptoms and signs, enhancements in quality of life and/or towards arresting/reversing neuronal degeneration (neuroprotection).

### 3.1. Mismatch between the Preclinical Data and the Clinical Data

In this study, our review has revealed a low successful translation rate from animal models to clinical assays. Interestingly, this phenomenon can also be seen in drug cancer research, where the average rate of translation from preclinical to clinical trials is less than 8% [118]. There could be multiple causes to our findings. In the section that follows, we will briefly discuss some of these causes.

There is a general acceptance that animal models have limitations for reproducing the complex pathophysiological mechanism present in Parkinson disease. There is no “perfect” animal model of PD; indeed, no translation has been achieve from the preclinical model to clinical neuroprotection in PD [119]. However, as most toxin-based lesion models (6-OHDA, MPTP, rotenone, etc.) act using similar pathways to those involved in the pathogenesis of PD, these toxins have been extensively used to evaluate neuroprotective agents [120]. In general, the use of toxin-based models, although providing a destruction of dopaminergic neurons and behavioral deficits, does not reflect the continuous “progressive nature” of pathogenic events occurring in humans. Neuroprotective intervention in humans must incorporate additional requirements in order to show cell recovery in target areas.

An alternate view is that animal models provide a translational result, but it is the method of subsequent clinical trials which are at fault or not reflective of any neuroprotection. Clinical essays are done mostly with large cohort of patients carrying mixed Parkinsonian pathology; hence, the detection of neuroprotection or disease-modification in some patients may be masked by others showing no neuroprotection. Patient selection for clinical trial is of extreme importance, paying particular attention to a sub-typing patients with PD based on genetics or biomarkers [120].

Other causes of the mismatch (Table 2):

### 3.2. Methodological Differences in Preclinical Experiments

What were the methodological points in the preclinical animal experiments that could have an impact in their predictive value? A key limitation in the evaluation of the preclinical studies is that there are so many different variables, for example in toxin dosage, neural center analyzed, animal models used, stages of treatment, and timeline of survival. These issues will be explored below.

Timing of the lesion: Some “pretreatment” studies start the neuroprotective intervention before a Parkinsonian state is established. In those studies, the intervention usually starts in a healthy animal and then the agents are applied either at the same time or even after the start of the therapeutics. This does not reflect the clinical situation where a PD diagnosis is usually made when 60–70% of the dopaminergic cells are degenerated. Administration of the NSI should begin once neurodegeneration has started or from a pre-defined level of loss of dopaminergic neurons to more closely mimic the clinical setting [7,122].

Striatal dopamine levels vs. SNc dopamine levels: The primary goal of a histological evaluation in a neuroprotective trial is to show that the subject treated with a NSI present considerably more dopamine and dopaminergic terminals in the striatum AND more surviving dopaminergic neurons in the SNc compared to control animals. In some studies, however, only striatal dopamine levels have been reported, with no assessment of either the effects of MPTP or the NSI on numbers of dopaminergic neurons in the SNc. An analysis of terminal density and cell number remains the key evidence for neuroprotection; they are also necessary to differentiate between terminal plasticity and neuronal protection [123].

Acute vs. Chronic: Some studies establish their lesions in an acute fashion. The acute model, although creating dopaminergic lesions and behavioral impairments, generally has the limitation of not being stable over time and can produce excessive morbidity. In addition, acute models do not reflect the natural evolution of idiopathic PD, where degeneration is a slow and continuous process in target areas [124]. Further, in some MPTP-treated models, there is evidence for spontaneous recovery in behavioral and striatal terminations [125], which can give the impression of improvement.

Rodent vs. non-human primate models: Most of the experimental studies have been undertaken in rodents, because they are more freely available for science, easier to handle and maintain and less expensive. This means that NSI are being tested in animals that are further phylogenetically from humans. Complex cell death mechanisms are also likely to be very different between rodents and primates. Non-human primates are a very sensitive to MPTP administration and produce a clear set of PD-like features, similar to those in humans [7]. A further important factor is the size of the animal. These therapeutics often require medical devices (pumps, pacemakers, etc.) to deliver the neuroprotective agent. For that, rodents need to have specially adapted equipment, which can introduce variability when translated to clinical trials; the final device could have technical problems undetected in preclinical essays (i.e., small implantable pumps will deliver the agent in target area, whereas bigger pumps could have problems of reflux).

Period of observation: Preclinical experiments differ in time of follow up. The majority of the studies are short- to medium-term, weeks to months. This means that agents that produce positive results over the short-term, may have the limitation of not being long-lasting. Longer periods of observation can also be useful in detecting any delayed onset side effects due to repeated applications of an agent or therapeutics. One example of this was seen in the preclinical experiments testing intraputaminal GDNF perfusion. Cerebellar toxicity was only identified when a 6 month long study was done using well-established Parkinsonian monkeys [117].

Methodological differences, however, do not change the fact that most of the preclinical experimental studies concluded that agents had positive potential for neurorestoration, but controlled clinical trials were not conclusive.

### 3.3. Clinical Trials Limits for Measuring Neuroprotection

Are there any appropriately designed clinical trials that show a neuroprotective effect? There are very few clinical trials using NSI which are really concerned with neurorestoration in an isolated fashion. In most cases, the main focus of the study was to obtain symptomatic benefit and better quality of life (See Table 3 for current indications of NSI). Neurorestoration is a phenomenon that difficult to disentangle from the symptomatic benefit of the therapies. Some studies, for instance, look at the integrity of the nigrostriatal pathway by using surrogate markers as striatal uptake of fluoro-dopa on positron emission tomography (PET) or β-CIT-on single-photon emission computerized tomography (SPECT) [8]. There is little evidence, however, that the uptake of these isotopes by the striatum is indeed correlated with the quantity of remaining dopaminergic neurons in SNc [8]. One of the newest strategies in neurorestoration measures in clinical trials is the delayed-start design trial. The concept of the delayed-start design trial was initially developed for Alzheimer’s disease patients, by Leber from the US Food and Drug Administration [126]. This design uses the total score on the well accepted UPDRS as the primary outcome measure. The patients are randomized to obtain therapy at time point T0 or to have it delayed for six or nine months until time point T1. By the end of the test, both patient groups will have received active treatment for 6–9 months, so any symptomatic effect should be equal in the two groups and any remaining difference must be due to a neuroprotective effect. However, the potential benefit of this study design [127] has raised questions about ethics and the possible overestimation of small differences in total UPDRS score which may not be clinically significant. As it stands, no clinical trials for putative neuroprotection in PD patients using NSI to date follow the delayed onset design model. They use the reversal of PD signs measured by the UPDRS as a principal end-point. This outcome measure is less than ideal because any symptomatic effect could be confounded with a neural restoration. To really separate this two effects, neuroprotection will require evaluation after a prolonged no treatment period. These “washout” periods also represent a limitation in most clinical trials, because it is very difficult and ethically questionable to plan a long period without symptomatic relief in an advanced PD patient. In addition, the length of the washout could raise some technical criticism: the residual effect or “carry-over effect” of the symptomatic drug treatment it is often very difficult to separate from potential neuroprotection [128].

Another limitation of the analysis of clinical trials is that most of the trials concerned patients with advanced PD. This is particularly true in the case of studies of DBS of the STN, where the main indication for surgery was motor fluctuation in advanced patients. Some early DBS STN essays are available, but with short periods of observation and usually no analysis of the baseline evolution of the patient during washout periods (Off stim/Off medication). In those published “early” studies, it is not possible to determine the degree of neurorestoration [130,135]. The challenge here is to include patients at the right time and avoid some of the justified criticism directed towards some early DBS STN studies (i.e., the risk of including patients too early, leading to the inclusion of atypical cases, plus the evident unjustified surgical risks) [136] with the obvious need to have functional reserves of dopaminergic cells to restore.

Finally, another issue with advanced PD patients is that they have prominent non-motor, non-L-Dopa sensitive symptoms in addition to classical motor signs [137]. There is an intrinsic limitation of most neurosurgical approaches for neuroprotection, which is the fact that they—for the most part—only target the dopaminergic system. Primary endpoints like overall quality of life and total UPDRS scores will still be unimproved with a therapeutic not targeting non-motor, non-Dopa sensible manifestations.

### 3.4. Recommendations for Increased Predictive and Translational Value of the Preclinical Animal Model and Trials

Ideally, NSI should be tested in new animals models that are more “pathophysiological based”. Such neuroprotection models should include several characteristics of the disease mechanism (Proteasomal dysfunction, chronic inflammation, mitochondrial?) as well as a progressive nigrostriatal degeneration [120]. Further, degeneration should incorporate non-dopaminergic regions. This ideal model is not yet available.

As no “perfect” animal model exists with all of the pathophysiological pathway characteristics, it will be instructive to test any given therapeutic intervention in several models—for example, from toxin-induced to transgenic—before translation to clinical trials. A full discussion concerning the development of new animal models is beyond the scope of this review; please see Duty et al. [120] for a complete survey.

Another key issue arising from our review is that therapies involving medical devices should be tested in larger animals. The animal and its related environmental and physiologic attributes should provide a system that offers a close simulation the clinical setting. The use of smaller versions of the final devices can introduce variability to the preclinical test. The FDA recommends that devices and delivery systems should be as similar as possible to the human model [138]. Customized versions of the device often comprise adapted and specific features suitable for the preclinical intervention requirements, whereas when passing into clinical trials, the new device has to be readapted, and standardized for make it suitable to clinical application.

Some authors have suggested changing the animal models’ experiment architecture. This has been proposed by Bezard for neuroprotective drugs, as the “clinically driven design” of preclinical studies. It has three criteria:

(1) The chosen animal model should recapitulate most, if not all, features of sporadic PD, including its progressiveness (all acute models are excluded, although useful for initial screening) and its pathological landmark (i.e., the replication of Lewy body like inclusions).
(2) Administration of the therapy should start only once a specific predefined level of dopaminergic degeneration has been achieved to better simulate clinical PD Always start the intervention once the degeneration of target dopaminergic neurons is advanced enough (60 to 70%). In this way, it will resemble the dopaminergic reserve of PD patients. This is common sense, yet some works have been performed in non-installed PD degeneration [124].
(3) Final proof of efficacy should be obtained from non-human primate models and not limited to rodents [7].

Other ideas related to experimental methodology could be in order to focus on neurorestoration, to adapt delayed onset trials to preclinical settings, mimicking neuroprotective clinical essays. New methodological avenues should be explored in order to filter the symptomatic effect, which tend to overestimate neuroprotection of the therapeutic intervention.

An important factor that could increase predictability is the use of longer periods of observation in chronic models. This model more closely follows the neuropathology seen in humans. In addition, this could allow researchers to observe the effects of the intervention in the long-term and detect late-onset side effects [117].

One key feature that has been overlooked is including the preclinical experiment additional elements present in the clinical trial like additional drug treatment. Very often, the negative outcome can also be related to factors not replicated or present in the preclinical animal experiments, for instance continuous therapeutic administration of L-Dopa or other drugs besides NSI. An example of this is the preliminary studies of mesencephalic fetal graft that failed to see late occurrence dyskinesia. Numerous rodent and MPTP-treated monkey experiments in which fetal mesencephalon was grafted into the striatum did not predict the common side effects seen in humans. None of the preliminary animal studies ever addressed the issue of potential interaction between grafted tissue and L-Dopa, and the animals were not treated at the same level as human patients. However, in recent experiments, some authors have been able to reproduce “runaway dyskinesia” in monkeys, simply by studying the interaction of L-Dopa and MPTP-chronic models [139].

## 4. Materials and Methods

Given the large quantity of published data, we approach the review in a systematic and selective fashion. Following Douna’s methodology for reviewing pharmacological neuroprotective agents [140], we performed a PubMed and Scopus search for “neuroprotective agents” AND “Parkinson disease”. From the list of therapies, pharmacological agents were excluded. Using the remaining “agents names”, we added “Neuroprotection” AND “Parkinson” to a new search and classified the resulting studies as preclinical or clinical. We then applied the following exclusion criteria:

- Studies that did not aim to provide neuroprotection were excluded
- Procedures with no published preclinical results were excluded
- Procedures not tested in clinical trials were excluded

This review identified 40 different types of NSI that can be grouped into three classes: Neuroprotection secondary to deep brain stimulation of subthalamic nucleus, Glial neurotrophic derived factor (GDNF) approach and cell grafting or transplant based therapies. Our discussion will be limited to such approaches that have been tested in clinical trials and we will compare previous preclinical results with final controlled clinical essays. In the Supplementary Materials, summary tables are provided for a more comprehensive overview of the clinical essays.

## 5. Conclusions

The current preclinical animal models of PD are perhaps more suited to reproduce phenotypic Parkinsonism and hence more suited to evaluate the symptomatic treatment of the disease. In this review, positive preclinical results in animal models were not particularly predictive of positive outcomes in randomized clinical trials.

Several aspects of the NSI methods used in animal models may be associated with this lack of success in clinical trials. One intrinsic limitation of most neurosurgical approaches for neuroprotection is that they predominantly target the dopaminergic system, leaving non-dopaminergic areas untreated. This is also the case for animal models, which usually lack that kind of degeneration. Some devices were scaled down in order to fit small animal models. This reduction in size could have been detrimental when extracting general conclusions prior to clinical trials. Hence, there is a need for the use of larger species when dealing with medical devices. Another important issue was the lack of preclinical indication of adverse clinical effects in humans (i.e., graft related dyskinesia in transplanted embryonic cells). One possible solution might be longer periods of observation and larger cohorts, where adverse effects may become clearer. In addition, other solutions might be an evolution in the preclinical models including more pathophysiological features (i.e., α-synuclein expressing models, as presented by Ip in a very recent paper showing Lewy body-like pathology after the induced overexpression of α-synuclein [141]), or repeated testing on several different preclinical models.

In summary, evaluation of neuroprotection has proved problematic in the clinical setting, mainly because the current clinical measures of neuroprotection are very limited; the “symptomatic effects” of the different approaches heavily mask any potential positive neuroprotective effects. It will be the challenge of future endeavors to develop new clinical measures of neuroprotection, perhaps with better developed functional imaging. At the end of this review, it is evident that none of the NSI approaches, although proving some preclinical efficiency, have provided a clinical benefit significant enough to lead to a systematically applicable neuroprotective strategy in human patients. The search for new neuroprotective avenues continues. Emerging neuroprotective therapies, such as near infrared photobiomodulation [142,143,144], are passing the preclinical stage, leading to upcoming clinical trials.

## Figures and Tables

**Table 1:** Clinical trials GDNF in PD.

Clinical Study	Type	No.	Therapeutics	Outcome	Side Effects
Nutt et al., 2003 [67]	Randomized placebo-controlled double-blind multicenter	50	GDNF-ventricular, monthly	UPDRS-III scores not improved	Nausea, anorexia, vomiting weight loss, paresthesia, hyponatremia
Kordower et al., 1999 [66]	Open label, safety study	1	GDNF-intraventricular catheter, monthly	worsen PD, no nigral recovery	Depression, anorexia, hallucination, nausea, sexual misconduct, tingling, Lhermitte phenomenon
Gill et al. 2003 [68]	Open label, safety study	5	GDNF-bilateral intraputaminal, continuous pump	57% improvement UPRDS-III off med	no serious side effects
Slevin et al., 2005 [69]	Open label, safety study	10	GDNF unilateral-intraputaminal, continuous pump	34% and 33% improvement UPRDS total score in on and off med	Lhermitte symptoms
Lang et al., 2006 [71]	Double blind multicentric randomized study	34	GDNF bilateral-intraputaminal, continuous pump	no significance placebo	2 repositioning + 1 removal device
Marks et al., 2008 [72]	Open label, safety study	12	GDNF-NTN bilateral-intraputaminal, AAV vector	36% UPRDS-III off med	no serious side effects
Marks et al., 2010 [73]	A double-blind, randomized, controlled trial	58	GDNF-NTN bilateral-intraputaminal, AAV vector	no significance to endpoint	3 tumors, serious adverse effects

GDNF: glial neurotrophic derived factor; PD: Parkinson’s disease; UPDRS-III: Unified Parkinson’s Disease Rating Scale part III; NTN: neurturin; med: medication.

**Table 2:** Causes of low successful translational rate of NSI from animal models to clinical data.

Animal Models	Clinical Trials
Due to intrinsic factors of the lesion models	Patients in the clinical studies have advanced Parkinson disease
Animal models experiment architecture focus in symptoms relief and not in restoration.	Clinical studies were not designed to measure neuroprotection
Publication bias: Seen in other areas like stroke research. Preclinical studies tend to overrate the positive outcome of a treatment by almost 30%, mainly because negative results are not reported [121]	

**Table 3:** NSI for neuroprotection: Causes of failure and Current indication.

NSI	Causes of Failure	Current Indication
DBS in STN neuroprotection:	Advanced PD patients. Confounding non dopaminergic symptoms. Off stimulation/off medication evaluation was not systematically performed. Not ideal dopaminergic imaging (PET, SPECT, etc.) to follow PD restoration in clinical trials. It is very difficult to quantitatively evaluate nigrostriatal pathway integrity during trials.	DBS STN is the standard alternative to motor fluctuation in advance Parkinson disease [129] and also recommended in some centers as an early intervention on Parkinson disease, not because of neuroprotective effect, but because its effects in the overall quality of life and avoidance of complications [130]. There is at best no consensus about the potential for neuroprotection of DBS STN [131].
GDNF Neuroprotection:	Technical Failure of delivery adequate quantity of growth factor in target area. Concern about possible late secondary effects, detected initially on primates: cerebellar degeneration was seen in NHP after infusion of putaminal GDNF [117]	GDNF INFUSION: There are no new clinical trials published after failed randomized controlled trials. There have been some communications explaining the failure as a technical issue due to reflux of the implantable pump, underlining the potential capacity of neurotrophic factors for neuroprotection [132]. New noninvasive approaches are being explored in preclinical trials, combining MRI Ultrasound to open BBB and intravenous application of GDNF [133].
Graft Neurorestoration:	Presence of side effects: violent dyskinesia related to ingestion of L Dopa [112]. This effects is due to excessive production of L Dopa locally and increase in DOPA receptors. Advanced Parkinson disease trials	BASAL GANGLIA GRAFTS: In theory, Adult stem cells and DA-graft in general would inhibit disease progression by secreting neurotrophic factors and by stimulating neuroplasticity beside simple DA secretion [134]. However, grafts remain largely experimental, with some teams still involved in developing new kinds of implantable cells like mesenchymal stem cells (MSCs) and hematopoietic stem cells (HSCs) [134]. As see in the review, clinical essays have only produces modest results

## Supplementary Materials

Supplementary materials can be found at www.mdpi.com/1422-0067/18/10/2190/s1.
