# Supplementary material for: Neuroprotective Surgical Strategies in Parkinson’s Disease: Role of Preclinical Data
Source: Int J Mol Sci. 2017 Oct 20;18(10):2190. doi: 10.3390/ijms18102190 (PMC5666871; doi:10.3390/ijms18102190)
Supplement: Supplementary file 1 [file ijms-18-02190-s001.pdf]

Supplementary Data: Additional clinical studies information for each evaluated intervention.

Table S1. Clinical Studies STN DBS long term evaluation.

Clinical Study	Type	No.	Follow Up (years)	Disease Duration (Years)	UPDRS-III off/off* (0–108)	UPDRS-III on/on** (0–108)	L-Dopa Reduction (mg/day)(LEDD)
Rodriguez et al., 2005	Long-term prospective evaluation Gpi vs. STN	49	4	15.4 ± 6.3	**Baseline**:56.7 ± 15.7 **1y**:53.7 ± 16.4 **4y**:54.8 ± 16.5	**1 y**:27.9 ± 17.2 **4 y**:29.5 ± 20.6	**Baseline**:1336 ± 619
Schüpbach et al., 2005	Long-term prospective evaluation	37	5	11 ± 1.0	**Baseline**:51.9 ± 17.2 **6m**:46.7 ± 19.2 **2y**:42.5 ± 15.6 **5y**:51.3 ± 15.4	**6 m**:11.3 ± 9.7; **2 y**:10.1 ± 9.9; **5 y**:17.9 ± 12.3	**Baseline**:1468 ± 811 **6 m**:559 ± 433 **24 m:**652 ± 448 **60 m**:667 ± 504
Visser-Vandewalle et al., 2005	Long-term prospective evaluation	20	5	no data	**Baseline**:42.3 ± 9.3 **4y**:53.6 ± 20.6	**3 m**:10.1 ± 5.9 **4 y**: 14.4 ± 7.5	47.2% from baseline at **4 y**
Østergaard et al., 2006	Long-term prospective evaluation	26	4	9 ± 5	**Baseline**:51.3 ± 12.1 **1y**:43.7 ± 13.1 **4y**:51.0 ± 19.4	**Baseline:**23.5 ± 15.8; **1y**:10.7 ± 7.2;**4y**:20.7 ± 11.6c	**Baseline**:1197 ± 532 **1 y**:964 ± 501 **4 y:**852 ± 590
Gervais-Bernard et al., 2009	Long-term prospective evaluation	23	5	12.9 ± 3.2	**Baseline**:43.11 ± 14.04; **1y**:41.5 ± 9.5 **5y**:45.9 ± 10.8	Baseline:14.83 ± 9.80; **1 y**:10.34 ± 5.47; **5 y:**13.17 ± 5.48	**Baseline:**1188 ± 465 **1 y:**333.7 ± 375 mg **5y:**509 ± 344 mg
Moro et al., 2010	Open. Non-randomized. prospective multicenter clinical trial and randomized double blind	49	6	13.5–15.3	**Baseline**:56.5; **3m**:54.0 ± 23.0; **5–6 y**:55.0 ± 23	**Baseline**:22.9 ± 2.0; **3 y**:18.6 ± 2.6; **5–6 y**:24.6 ± 2.9	LEDD was not significantly reduced at 5 to 6 years
Tagliati et al., 2010	Long-term retrospective evaluation	50	5	11 ± 1.0	**Baseline**:40.1 ± 13.8; **1y**:38.1 ± 13.1; **2y**:39.3 ± 13.6; **3y**:43.3 ± 14.9 4 y:40.9 ± 14.1; **5y**:42.1 ± 14.5	no data	Baseline:1054 ± 506; **1 y**:773 ± 528 **2 y**:789 ± 586 **3 y**:556 ± 275 4 y:796 ± 778 **5 y**:715 ± 411
Castrioto et al., 2011	Class III study: Prospective open label blind evaluated	18	10	13.4 ± 4.8	**Baseline**:50.2 ± 13.3; **1y**:45.21 ± 11.8; **5y**:49.3 ± 11.7; **10y**:49.5 ± 10.3; **10y**:48.6 ± 11.7;(blinded evaluation)	**Baseline**:50.2 ± 13.3 **10y**:35.0 ± 13.1(blinded evaluation)	Baseline:1237.8 ± 547; **1 y**:665.6 ± 311.4; **5 y**:705.6 ± 272.5; **10 y**:788.9 ± 485.5

Merola et al., 2011	Long-term retrospective evaluation	19	8.42	22.84 ± 2.29	**Baseline:**61.40; **7 y**:63.23	**Baseline**: 18.25; **5 y**:26.38; **7 y:**27.57	**Baseline:**890.38 ± 299; **1 y**:336.50 ± 189.30; **3 y**:381.63 ± 180.46; **5 y**:488.90 ± 265.50; **7 y**:435.10 ± 223.20
Zibetti et al., 2011	Long-term prospective evaluation	14	9	17.0 ± 4.7–16.0 ± 5.1	**Baseline**:51.3 ± 15.4; **1 y**:54.4 ± 10.9;**5y:**54.9 ± 12.8; **9y:**56.3 ± 18.4	**Baseline:**16.5 ± 11.0; **1 y**:11.4 ± 6.8; **5 y**:17.4 ± 8.1; **9 y**:23.4 ± 11.0	**Baseline**:955 ± 406 **1 y:**412 ± 265 5 y:569 ± 288 **9 y**:579 ± 295
Merola et al., 2013	Comparative retrospective medical DBS vs. STN	19	6	12 ± 1	**Baseline**:45.37 ± 8.09; **6 y**:53.47 ± 16.81	**Baseline:**14.79 ± 4.40; **6 y**:24.97 ± 14.16	**Baseline:** 935.6 ± 21.5 **6 y**:572.6 ± 19

STN: Subthalamic nucleus; DBS: Deep brain Stimulation; UPDRS-III: Unified Parkinson's Disease Rating Scale part III; LEDD: levodopa equivalent daily dose; **y:** years**; m:** months; No: number of patients; *:UDPRS score off stimulation and off medication; **: UDPRS score on stimulation and on medication

Table S2. Clinical studies in cell grafting or transplant based therapies for Parkinson's disease.

Clinical Study	Type	No.	Therapeutics	Outcome	Side effects	Dose/Immunosuppre ssion
Backlund et al., 1985	Open label	2	Autologous adrenal medullary tissue: transplanted to the striatum unilaterally	Px 1:no change in scores, Px 2:slight worse after 2 years	Psychiatric symptoms (paranoia)	15 pieces 2–3mm
Lindvall et al., 1987	Open label	2	Autologous adrenal medullary tissue: transplanted to the striatum unilaterally	Px 3: 6month improvements then no change Px 4: no change	no data	15 pieces 2–3mm
Madrazo et al., 1987	Open label	2	Autologous adrenal medullary tissue: transplanted to the striatum unilaterally	Rigidity and akinesia: disparition, tremor reduced: UPDRS off state reduction at **3y** in Px1: 33%,and in Px2: 46%	no data	
Lindvall et al., 1989	Open label	2	Human fetal dopamine neurons : transplanted to the striatum unilaterally	Modest clinical change. Increase response to Dopa. F-Dopa PET: No changes	no data	8 to 10 weeks age embryo/
Lindvall et al., 1990	Open Label	1	Human fetal dopamine neurons : transplanted to the striatum unilaterally	Off state changes in bradykinesia. Increase response Dopa. F-Dopa PET increase uptake :130%	no data	8 to 10 weeks age embryo/ Immunosuppression
Tanner and United Parkinson Foundation Neurotransplantation Registry Group, 1991	Multicentric open evaluation	61	Autologous adrenal medullary tissue : transplanted to the striatum	UPDRS "off" state reduction at 2y: 19% in all Px	18 surgical related dead, 22% psychiatric morbidity	
Henderson et al., 1991	Open Label	12	Human fetal dopamine neurons : transplanted to the striatum unilaterally	3 Px responded: 61%reduction levodopa 1 year	3Px deteriorated	11 to 18 weeks age embryos / Immunosuppression not used.

Freed et al., 1992	Open Label	7	Human fetal dopamine neurons: transplanted to the striatum bilaterally	UPDRS "on" state reduction in 6 Px: 39%, Hoen Yang score reduction 1.21 ± 0.26	no data	7 to 8 weeks age embryos / Immunosuppression
Piccini et al., 2000	Open Label	4	Human fetal dopamine neurons: transplanted to the striatum bilaterally	F-Dopa PET increase uptake at 6,5 month, UPDRS "on" state reduction at 18 month: 50%; L-dopa reduction: 60%	no data	5–7 weeks age embryos; length of 13–27 mm from crown to rump/immunosuppres sion
Iacono et al., 1992	Open Label	5	Human fetal dopamine neurons: transplanted to the striatum bilaterally			
Lindvall et al., 1992	Open Label	2	Human fetal dopamine neurons : transplanted to the striatum unilaterally	reduction of the time spent in the "off"state and the number of daily "off" periods; a lessening of bradykinesia and rigidity during the "off" state	no data	6–7weeks age embryos/ immunosuppression
Spencer et al., 1992	Randomized delayed onset study	4	Human fetal dopamine neurons : transplanted to the striatum unilaterally	3 Px improve. Lower dose Dopa in 1Px. F-Dopa PET increase uptake in 1Px	lack serious side effects	7 to 11 weeks age embryos /Cyclosporine was administered for six months postoperatively.
Widner et al.,1992	Open Label	2	Human fetal dopamine neurons: transplanted to the striatum bilaterally	Levodopa was decrease 30% in 1Px; F-Dopa PET: increase uptake in all Px	no serious complications	six to eight weeks age embryos /immunosuppression
Freeman et al., 1995	Open Label	4	Human fetal dopamine neurons: transplanted to the striatum bilaterally	Total UPDRS 'off' state reduction: from80.3 ± 8.3 to 58.0 ± 10.5; Schwab-England score off: from 51.3 ±5.2 to 72.5 ± 1.4; F-Dopa PET: increase uptake of 53% on the right, 33% on the left	Asymptomatic superficial cortical hemorrhage, transient postoperative confusion and hallucinations.	6 1/2 to 9 weeks age embryos/ immunosuppression for 6 month
Defer et al., 1996	Open Label	5	Human fetal dopamine neurons: transplanted to the striatum unilaterally	Long-term bilateral improvement of skilled hand movements. A mild to moderate effect on the amount of 'off' time and 'on-off' fluctuations was observed, whereas, apart from one case, no clear effect on gait, walking and speech. L-dopa therapy at a similar or higher dose than before	Dementia in 1Px Delayed asymmetrical dyskinesia in 3Px	
Wenning et al., 1997	Open Label	6	Human fetal dopamine neurons: transplanted to the striatum unilaterally	F-Dopa PET: 68% increase uptake Clinical Improvement in 4 Px	No consistent changes in dyskinesias	

Hagell et al 1999	Open Label	5	Human fetal dopamine neurons: transplanted to the striatum bilaterally(sequencially)	F-Dopa PET: 85% increase uptake	Off medication dyskinesia in long term evaluation. Atypical features, who responded poorly to the first graft, worsened following the second transplantation	four to eight donors (6–8 weeks age embryos)/immunosuppression
Hauser et al., 1999	Open Label	6	Human fetal dopamine neurons: transplanted to the striatum bilaterally	UPDRS "off" state reduction :32%; time spent in "on" state without dyskinesia: improved from 22% to 60%; F-Dopa PET: increase uptake	Two patients died: unrelated surgery	6 1/2 to 9 weeks age embryos /immunosuppression
Freed et al., 2001	randomized double blind sham surgery	40	Human fetal dopamine neurons: transplanted to the striatum bilaterally	F-Dopa PET: increase uptake in 17 Px	dystonia and dyskinesia in "off "state in 15 percent of the patients 3 years after graft	
Olanow et al., 2003	randomized double blind sham surgery	34	Human fetal dopamine neurons: transplanted to the striatum bilaterally	There was no significant overall effect UPDRS "off" state. F-Dopa PET increase uptake in grafted groups at 24 month	56% Px with off-medication dyskinesia	
Mínguez-Castellanos et al., 2007	open label phase I-II	13	Carotid body (CB) glomus cells: transplanted to striatum bilaterally	UPDRS-III reduction: at 6 m:23%, 1 y: 5–74%, 3y: 15–48%. F-Dopa PET: non-significant 5% increase uptake	No data	autologous CB graft/no immunosuppression
Kordower et al., 2008	neuropato study	1	Human fetal dopamine neurons: transplanted to the striatum bilaterally		Lewy body inclusion in grafted nigral neurons	
Li et al., 2008	neuropato study	2	Human fetal dopamine neurons: transplanted to the striatum bilaterally	11–16 years evolution	Lewy body inclusion in grafted nigral neurons	
Venkataramana et al., 2010	open label phase I-II	7	BM-MSC: transplantation in sub-lateral ventricular zone unilaterally	UPDRS "off" state reduction: 22,9%; UPDRS "on" state reduction: 38%	no adverse effects	1 million cells/kg body weight of BM-MSC/ no immunosuppression
Venkataramana et al., 2012	open labelI-II	12	BM-MSCs: transplantation into the subventricular zone bilaterally	UPDRS "off" state reduction: 32%; UPDRS "on" state reduction: 17.92%; clinical improvement: clarity in speech, reduction in tremors, rigidity, and freezing attacks.	Parkinson plus no improvement	1 million cells/kg body weight of BM-MSC/ no immunosuppression

UPDRS: Unified Parkinson's Disease Rating Scale; BM-MSC: Autologous bone-marrow-derived mesenchymal stem cell; F-Dopa PET: fluoro-3,4-dihydroxyphenylalnine positron emission tomography Px: Patient; No: number of patients.
